# Synergistic Structure in the Speed Dependent Modulation of Muscle Activity in Human Walking

**DOI:** 10.1371/journal.pone.0152784

**Published:** 2016-04-01

**Authors:** Tom J. W. Buurke, Claud J. C. Lamoth, Lucas H. V. van der Woude, A. Rob den Otter

**Affiliations:** Center for Human Movement Sciences, University Medical Center Groningen, University of Groningen, Groningen, The Netherlands; Rutgers University -New Jersey Medical School, UNITED STATES

## Abstract

Recently, a modular organisation has been proposed to simplify control of the large number of muscles involved in human walking. Although previous research indicates that a single set of modular activation patterns can account for muscle activity at different speeds, these studies only provide indirect evidence for the idea that speed regulation in human walking is under modular control. Here, a more direct approach was taken to assess the synergistic structure that underlies speed regulation, by isolating speed effects through the construction of gain functions that represent the linear relation between speed and amplitude for each point in the time-normalized gait cycle. The activity of 13 muscles in 13 participants was measured at 4 speeds (0.69, 1.00, 1.31, and 1.61 ms^-1^) during treadmill walking. Gain functions were constructed for each of the muscles, and gain functions and the activity patterns at 1.00 ms^-1^ were both subjected to dimensionality reduction, to obtain modular gain functions and modular basis functions, respectively. The results showed that 4 components captured most of the variance in the gain functions (74.0% ± 1.3%), suggesting that the neuromuscular regulation of speed is under modular control. Correlations between modular gain functions and modular basis functions (range 0.58–0.89) and the associated synergistic muscle weightings (range 0.6–0.95) were generally high, suggesting substantial overlap in the synergistic control of the basic phasing of muscle activity and its modulation through speed. Finally, the combined set of modular functions and associated weightings were well capable of predicting muscle activity patterns obtained at a speed (1.31 ms^-1^) that was not involved in the initial dimensionality reduction, confirming the robustness of the presently used approach. Taken together, these findings provide direct evidence of synergistic structure in speed regulation, and may inspire further work on flexibility in the modular control of gait.

## Introduction

For the production of bipedal human gait, more muscles are available than are necessary to produce stepping, and this ‘degrees-of-freedom’ or ‘redundancy’ problem has intrigued researchers since it was first introduced by Nikolai Bernstein[1]. At the same time, the redundancy implied in the controlled system offers abundant possibilities to modify the basic locomotor pattern allowing the performance of functionally coherent movements and the accommodation to continuously changing task and environmental demands. As such, ‘complexity’ of control and behavioral ‘flexibility’ can be interpreted as inextricably connected and should eventually be explained within one unifying framework[2]. In recent years, it has been suggested that the Central Nervous System (CNS) may overcome redundancy in the locomotor system by using a single, simplified form of neuromuscular control to be employed in a broad range of task contexts[3–5]. In this approach, motor outputs are generated through activation of groups of muscles that are driven by the combined activity of a small set of control signals, so called ‘modules’. Because these signals do not activate muscles individually, but instead simultaneously drive muscle groups that are functionally related, called ‘synergies’, a dramatic reduction in the complexity of control can be achieved[6,7]. However, how this type of low-dimensional neuromuscular control can be flexibly recruited to give rise to the rich and adaptive repertoire of human stepping behaviors is still not well understood.

Arguably one the most fundamental adaptive mechanisms in human gait is the ability to modify the speed of progression, e.g. to accommodate time pressure or to enhance safety. To propel the body forward, gravitational potential energy of the center of mass (CoM) is partly transformed into kinetic propulsive energy with a speed-dependent efficiency[8,9]. The CoM momentum is controlled by activity from the calf muscles, determining the changes in step length and/or cadence that are required to alter the speed of progression[10]. At the neuromuscular level these changes are controlled by phase-specific modulations in EMG amplitude that are superimposed upon a basic activation pattern[11–17]. These adaptations in neuromuscular activity serve to control the propulsive force, the altered accelerative properties of the swinging leg, and the speed-related changes in leg loading and support demands.

If human walking is governed by a single modular architecture, the basic phasing of muscle activity and its adaptive control of speed need to be realized within a single modular control scheme. Indeed, there is evidence from a number of studies showing that the synergistic structure, and the modular activation signals it drives, are essentially preserved over gait speeds[18–21]. In these studies, the invariance of modular control is tested by recording electromyographic signals (EMG) from a set of muscles over a range of speeds, and applying pattern recognition methods to reduce the dimensionality (e.g. non-negative matrix factorization, PCA, or factor analysis), for each speed condition separately[18–21]. These procedures result in a sparse set of modular activation functions that represent the temporal properties of modular control signals, and a set of associated synergistic weightings that reflects how these control signals are distributed over individual muscles. To evaluate invariance of modular control over speeds, modular activation patterns and synergistic weightings are then compared between speed conditions. The proposed modular mechanisms responsible for the neuromuscular regulation of speed involve adjustments made to synergistic weightings[18–20], rescaling of modular activation signals[21], and shifts in a speed-dependent phase factor to account for timing differences in modular activations[19,20]. Results from these studies convincingly show that variance in the activity of large sets of muscles can be accurately captured using a small set of 4 or 5 modular activations, and that the timing properties of these activations show clear resemblances between speeds. Although these findings indicate that a single modular structure may be involved in the control of walking over a range of speeds, they may not provide direct evidence for the idea that the dynamic adjustments made to muscle activity for speed regulation are under modular control.

As EMG patterns at a given speed contain both (i) amplitude variation related to speed-independent (i.e. basis) activity of the muscle and (ii) amplitude variation that is uniquely related to the speed at which the EMG is recorded[13], it remains unclear to what extent the previously reported dimensionality reductions are the result of covariance in the speed-independent basis activity of muscles, or of speed-dependent modulations in the basis-activity (see [22] for a similar argument). As a result, the existing literature[5,18,20] provides indirect evidence for the idea that the phase specific modulation of muscle output amplitude that is involved in speed regulation, is under modular control. Arguably, more direct evidence of the modular properties of speed regulation would involve a dimensionality reduction that specifically targets these speed effects, independent of the basis activity of muscles.

Because the amplitude of muscle activity scales linearly with speed, the phase-dependent modulation of the amplitude can be described using gain functions[12,13,23]. These gain functions represent, for each moment in the time-normalized gait cycle, the linear relation between muscle activity amplitude and speed. In a study by Hof and colleagues[13], gain functions were used to successfully model recorded EMG signals over a range of speeds (0.75 to 1.75 ms^-1^). An important finding from this study was that there were clear similarities in the basic activation of muscles as well as in their gain functions, so that an initial set of 13 basic patterns and 13 gain functions could eventually be reduced to a compact set of 6 basic muscle activation patterns and 10 related gain functions. These findings are important as they suggest that both the basic phasing of activity, as well as its modulation by speed are subject to modular control. In the present study, we elaborate on these ideas, and further explore the modular aspects of speed related changes in muscle activity during human walking. More specifically, the aims of this study were threefold. First, we assessed if speed related neuromuscular gain is subject to modular control. To this end, gain functions were determined for a group of muscles over a range of speeds. Subsequently, these gain functions were subjected to dimensionality reduction to determine if speed effects can be described using a parsimonious set of modular gain functions, indicating synergistic structure in the neuromuscular regulation of speed. Second, we determined whether the basis activation of muscles and the speed-related gains are driven by a single modular control structure. For this purpose, dimensionality reduction was performed on the muscle activations at a single walking speed (1.00 ms^-1^), to obtain a set of modular basis functions and their associated synergistic weightings. Next, the modular basis functions and modular gain functions, as well as their associated synergistic weightings, were compared to assess their similarities. Finally, to test the robustness of the present approach, we established whether the combined set of modular basis functions and modular gain functions, and their associated synergistic weightings, can be used to predict muscle activation patterns at a speed that was not involved in the dimensionality reductions.

An important requirement for the prediction of speed-related variations in muscle activity from a set of modular activations and synergies, is that speed needs to be used as an input to the proposed modular architecture, and that it therefore needs to be made explicit which aspect(-s) of the modular architecture (e.g. the scaling of synergistic weight, phasing of modular activations, shape characteristics of modular activations, etc.) are affected by speed. Recently, Gonzalez-Vargas and co-workers[18] have shown that it is possible to produce accurate predictions for a range of speed and elevation conditions, by scaling the synergistic weightings that drive an invariant set of 4 modular activations. Such predictions can be very useful for testing ideas on flexibility within a modular framework, and for providing evidence for the robustness of the proposed approach.

## Materials and Methods

### Participants

Thirteen volunteers (6 males, 7 females, 21.8 ± 2.2 years old, body height: 1.79 ± 0.07 m, body weight: 71.3 ± 9.3 kg) without any known physical or neurological impairments participated in this study.

### Ethics statement

The procedures of this study were approved by the Ethics Committee of the Center for Human Movement Sciences, University Medical Center Groningen, the Netherlands, and were in accordance with the principles outlined in the Declaration of Helsinki [24]. All participants gave their written informed consent.

### Experimental protocol

Participants walked on a treadmill (Enraf-Nonius, Rotterdam, the Netherlands) with a walking surface of 1.5 m long by 0.5 m wide at four different gait speeds (1.61, 1.31, 1.00, and 0.69 ms^-1^). Prior to testing, participants walked on the treadmill for five minutes to adjust to the treadmill. At the start of each trial, participants walked on the treadmill for thirty seconds to adjust to the new speed before testing commenced. Data for each speed condition were collected for one minute, and speeds were presented in the same quasi-randomized order of gait speed conditions (1.31, 1.61, 0.69, 1.00 ms^-1^) for all participants. No instructions were given with regard to stride length or cadence.

### Data recording

EMG data was recorded using a Porti EMG unit and Portilab2 software (TMSI, Enschede, The Netherlands) at a sample frequency of 2048 Hz. Electrodes were placed unilaterally on eleven lower extremity muscles (Soleus (SO), Gastrocnemius Lateralis (GL), Tibialis Anterior (TA), Peroneus Longus (PL), Vastus Lateralis (VL), Rectus Femoris (RF), Biceps Femoris (BF), Semitendinosus (ST), Adductor Magnus (AM), Tensor Fascia Latae (TFL), Gluteus Maximus (GM)), and two back muscles (Erector Spinae (ES), and Latissimus Dorsi (LD)). Electrodes were placed according to SENIAM conventions[25], and according to Perotto[26] if a muscle was not included in SENIAM (AM, LD). Body hair was removed, and the skin was abraded and cleaned with alcohol to improve conduction. Custom-made insoles with pressure sensors (three under the forefoot, one under the heel) were used to determine initial contact and swing onset. The pressure sensors were connected to the EMG unit’s auxiliary ports and the EMG and sensor data were synchronized and stored on an external hard drive for further (offline) analysis.

### Data processing

EMG and pressure sensor data was processed using custom-made software routines in MATLAB (version r2015a; The MathWorks Inc., Natick, MA). EMG data was high pass filtered using a 10Hz second order Butterworth filter, then rectified, and finally low pass filtered using a 10Hz second order Butterworth filter. Next, EMG data was normalized in the time domain for stance phase (65 data points) and swing phase (35 data points) separately, to a total of 100 data points per stride. To be able to estimate the linear relationship between amplitude and speed for each moment in the normalized gait cycle, accurate temporal alignment of the EMG patterns is paramount. If ‘regular’ stride normalization would have been used (i.e. from initial contact to initial contact) this may lead to misalignment of functional gait phases (e.g. t = 65% may correspond with the early swing phase at higher speeds and with late stance at slow speeds). An important consequence of this choice is that the phase shifts in modular activation patterns that are typically reported for different speeds [19,20] will be strongly attenuated or neutralized. Finally, the time normalized EMG signals were normalized for amplitude by dividing the EMG amplitude by the maximum amplitude over all speed conditions, for each muscle. Average EMG profiles were then calculated for each muscle and condition, resulting in a 13 (muscles) by 4 (speeds) by 100 (datapoints) matrix, for each participant. This matrix was then subjected to further analysis. On average 34 ± 2.5 strides were available for analysis per subject and speed condition.

#### Calculation of gain functions and gain modules

**Fig 1:**
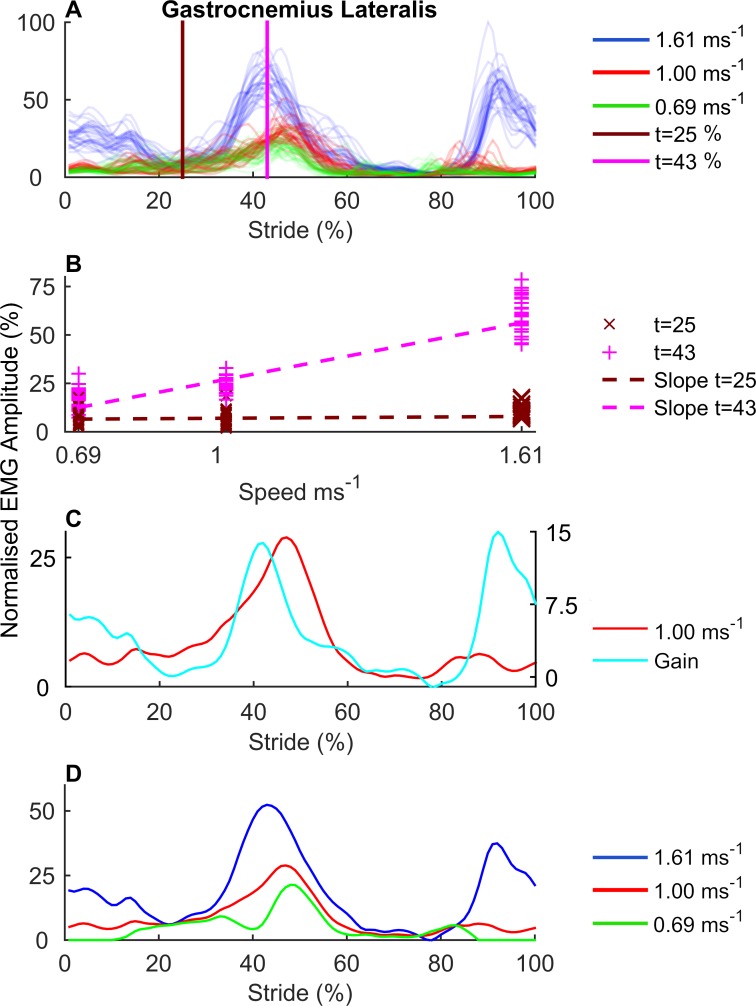
Calculation of gain functions. (A) Time and amplitude normalized EMG signals for individual strides (n = 30 per speed) of the Gastrocnemius Lateralis (GL), for a single participant at 0.69, 1.00, and 1.61 ms^-1^. The lines at t = 25% and t = 43% mark the points in the time normalized stride cycle which are illustrated more elaborately in ***Fig* 1B** (B) The linear relation between gait speed and EMG amplitude illustrated for t = 25% and t = 43% in the time normalized stride. For both time instants, a line is fitted to model the relation between speed and amplitude, using linear approximation. The slopes of the resulting linear approximation represents a gain factor that reflects the increase in EMG amplitude per unit increase in gait speed (ms^-1^). The figure illustrates that for t = 43%, the amplitude of GL activity increases with speed, whereas for t = 25% EMG amplitude is unaffected by speed. By calculating the gain factor for each time instant t(1…100) in the time-normalized gait cycle, a gain function can be constructed for each muscle. Figure (C) shows the gain function for GL and the averaged GL activity for this participant at 1.00 ms^-1^, and illustrates that speed effects were mainly present within a specific phase (approx. between 35% and 50%) of the main burst of GL activity. (D) Reconstructed EMG profiles at 0.69, 1.00, and 1.61 ms^-1^. As the gain function represents the linear increase in EMG amplitude per unit speed, the EMG profile for each muscle can be reconstructed for any given speed, using (i) the gain function for that muscle and (ii) the averaged EMG profile for that muscle at a given speed. ***Fig* 1D** shows the reconstructed GL profiles at 0.69 and 1.61 ms^-1^ using the GL gain function and the averaged activity at 1.00 ms^-1^.

Our first aim was to assess if speed related neuromuscular gain is subject to modular control. In general, muscle output amplitude scales approximately linearly with speed[13], although for specific muscles this may not be the case. For example, in Rectus Femoris activity around the stance-swing transition often increases suddenly at a critical speed[27] and Semitendinosus selectively shows marked activity during this period only at very low speeds[12]. Although such non-linearities cannot be captured in the here calculated gain functions, linear approximations of the speed-amplitude relationship are known to produce accurate reconstructions of EMG patterns over a range of speeds[13,23]. To this end, for each of the *m* (1…13) muscles, a gain function *G* was calculated that represents the linear dependency of the normalized EMG amplitude on speed, for each point *t* in the time normalized gait cycle[12,13]. Construction of the gain functions is illustrated in **Fig 1**. First, the first 30 strides for each speed condition were selected for further analysis (**Fig 1A**). Note that for an un-biased estimate of the linear relation between speed and amplitude, an equal number of strides has to be available for each speed condition. Gain functions were calculated by performing linear approximation of EMG amplitude on speed, for each time instant *t* (1…100) in the time normalized gait cycle (see **Fig 1B**). The gain was defined as the slope of the resulting linear approximation equations, and represents the increase in EMG amplitude (μv) per unit increase in speed (ms^-1^). Performing this operation for each *t* resulted in a gain function *G*_*m*_ for each muscle *m* (**Fig 1C**).

To assess whether the gain functions provided an accurate representation of the speed effects on EMG profiles, the averaged EMG profile of muscle *m* (1…13) was predicted from the gain function *G*_*m*_ and the averaged EMG profile of *m* at 1.00 ms^-1^, for each speed *v* (0.69, 1.31, and 1.61 ms^-1^, see **Fig 1**D), and for each participant, as follows:

$${\tilde{EMG}}_{v,m}={EMG}_{1.00,m}+(v\;*\;G_{m})$$ (1)

To assess the modular properties of the gain functions, modular gain functions were calculated for each participant. To this end, the gain functions *G*_*m*_ were subjected to principal component analysis (PCA) followed by varimax rotation[19,28]. This method was preferred to other methods often used, such as non-negative matrix factorization, because gain functions may contain negative values if EMG amplitude decreases with increasing speed. However, both methods generally produce similar results[5]. One of the aims of this study was to compare modular gain functions and the associated synergistic muscle weightings with modular basis functions and weightings obtained from the EMG profiles recorded at 1.00 ms^-1^ (see next section). To do so, the number of components that was extracted for the modular gain functions and modular basis functions had to be equal and needed to be defined a priori. In accordance with previous studies[18,21], the number of components that was extracted was set to 4. On average, the modular basis functions accounted for 80.1% of the variance in the original data set. In previous studies a variance accounted for > 80% was regarded sufficient for this analysis[29,30]. This was confirmed through further analysis, showing that an additional fifth modular basis function accounted for 5.8% of variance. Since this is less than the variance accounted for by a single muscle (7.7%), a fifth modular basis function was left out in this model. The percentage in the gain functions of individual muscles *m* that was explained by the modular gain functions was assessed to evaluate the extent to which speed related neuromuscular gain is subject to modular control.

#### Comparison of modular gain functions and modular basis functions

**Fig 2:**
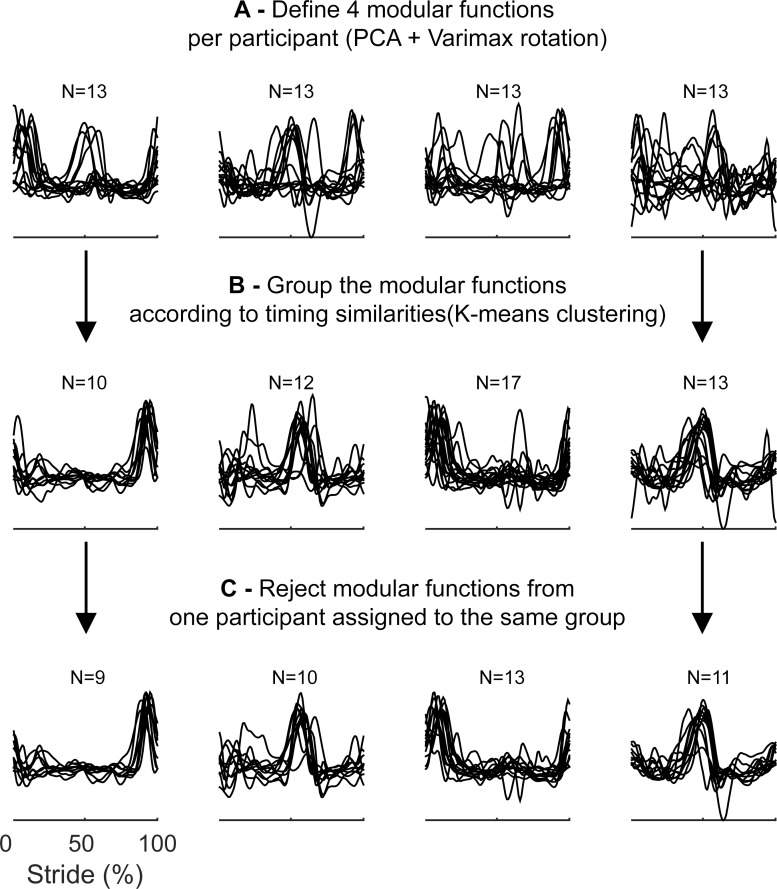
Grouping and selection of modular functions. Grouping and selection of modular functions was done in 3 steps. In step 1 (**Fig 2A**), 4 modular functions are constructed using PCA with Varimax rotation, for all 13 participants individually. In step 2 (**Fig 2B**) the modular functions are clustered according to their timing similarities, using K-means clustering. If the clustered functions contained more than one function from a single participant, in step 3 (**Fig 2C**), only the modular function with the smallest distance to the cluster centroid was retained, the other function (-s) within the cluster was/were rejected. Rejected gain functions were not re-assigned. Therefore, the number of functions within each cluster was typically smaller than the number of participants (i.e. n = 13). The same workflow illustrated here was used for the modular basis and modular gain functions.

The second aim of this study was to determine whether the basis activation of muscles and the speed-related gain are driven by a single synergistic control structure. To this end, modular gain functions and their associated muscle weightings were compared with modular basis functions and synergistic muscle weightings obtained after dimensionality reductions of the averaged activity patterns at 1.00ms^-1^. PCA with varimax rotation was applied to the set of 13 averaged muscle activation patterns at this speed, for each participant, to obtain modular basis activations and their associated synergistic muscle weightings. After extraction of the modular gain functions and modular basis functions for individual participants, modular functions were matched and grouped so as to allow meaningful averaging of functions and comparisons between both sets of averaged functions and their associated synergies. The grouping procedure is illustrated in **Fig 2**.

The varimax rotated PCA typically extracts modular functions in the order of the amount of variance they explain (i.e. function 1 explains the most variance, function 4 the least amount of variance in the original data set), but modular functions with similar timing characteristics are not necessarily extracted in the same order for different participants, as is shown in **Fig 2A**. Therefore, modular functions were grouped over participants according to their timing similarities (**Fig 2B**), so as to obtain homogeneous groups of modular functions and allow meaningful calculation of group averaged modular functions (see [31,32] for a similar approach). Here, we used K-means clustering[33] as a non-supervised method to group modular functions between participants. The number of clusters was set a priori to 4, to correspond with the number of components extracted by the PCA, so that the clustering procedure partitioned the (4 modules x 13 participants) 52 modular functions into 4 clusters. In case two modular functions of a single participant were assigned to the same cluster, the modular function with the smallest distance to the cluster centroid was assigned to that cluster, whereas the other modular function was rejected (**Fig 2C**). As a consequence, the number of individual modular functions assigned to a cluster could vary per cluster. This grouping procedure was applied separately for the modular gain functions and the modular basis functions.

To determine if the basis activation and speed-related gain share a similar modular structure, correlations were calculated between the cluster means of the modular basis functions and the cluster means of the modular gain functions, to obtain pairs of modular basis and gain functions with similar temporal properties. The magnitude of these correlations was interpreted as an indication of the similarity between modular gain functions and modular basis functions. To assess the similarity between the associated synergistic muscle weightings of both sets of functions, the averaged muscle weightings were calculated for each of the clustered sets of modules, for both gain modules and basis modules. Correlations between both sets of synergistic muscle weightings were used to assess their similarity.

#### Reconstruction and prediction of EMG using modular gain and basis functions and associated weightings

We tested the robustness of the presently used approach by reconstructing the averaged EMG profiles of individual participants using the combined set of 4 basis and gain modular activations and the associated weightings. The averaged EMG profiles at *v* = 0.69, 1.00, and 1.61 ms^-1^ were reconstructed as follows:

$${\tilde{EMG}}_{v,m}=H_{basis}W_{basis}+v(H_{gain}W_{gain})$$ (2)

*H*_*basis*_ and *H*_*gain*_ represent the 4 by 100 matrix of modular basis and gain functions respectively, *W*_*basis*_ and *W*_*gain*_ represent the 4 by 13 matrix of associated synergistic muscle weightings, and *v* represents speed (ms^-1^).

This method was also used to achieve the third aim of this study. To determine if the combined set of modular basis and gain functions, and their associated weightings, could be used to predict muscle activation patterns at a speed that was not involved in the dimensionality reductions, EMG at *v* = 1.31 ms^-1^ was predicted.

The amount of variance in the averaged EMG profiles that was accounted for by the reconstructions and prediction was used to assess the reconstruction and prediction quality.

To assess how well the modular gain functions can predict novel data of different subjects, we applied a bootstrapping procedure. For each of the n = 100 runs of the bootstrapping procedure, we calculated how well n = 7 randomly selected subjects were capable of predicting speed-related gain of each of the remaining n = 6 subjects. In each run, the modular gain functions of each of the 7 randomly selected subjects were used to obtain a set of 4 averaged modular gain functions. These functions were then combined with the modular basis functions of each of the remaining 6 subjects, to predict the average EMG profiles of each of these 6 subjects individually, for each muscle and speed (see Eq 2). Finally, the predicted profiles were averaged over the group of 6 subjects. The average variance accounted for (with 95% confidence interval) by the predictions was calculated for each speed condition, and averaged over all 100 bootstrap runs.

## Results

**Fig 3:**
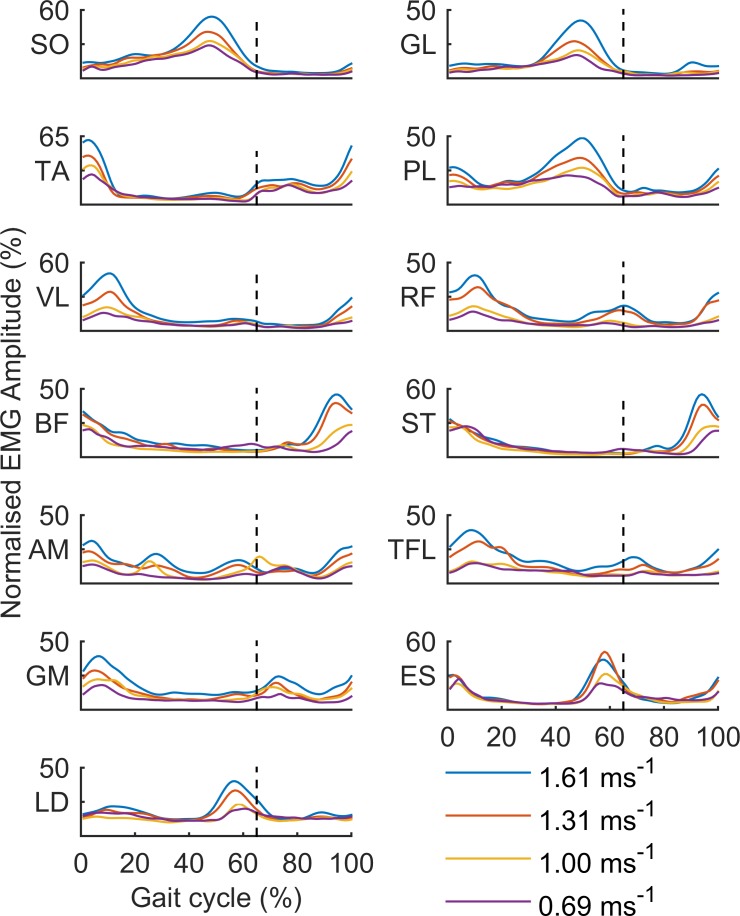
Group averaged EMG profiles at 0.69, 1.00, 1,31, and 1.61ms^-1^ for Soleus (SO), Gastrocnemius Lateralis (GL), Tibialis Anterior (TA), Peroneus Longus (PL), Vastus Lateralis (VL), Rectus Femoris (RF), Biceps Femoris (BF), Semitendinosus (ST), Adductor Magnus (AM), Tensor Fascia Latae (TFL), Gluteus Maximus (GM)), (Erector Spinae (ES), and Latissimus Dorsi (LD). The dotted lines represent swing onset at 65% of the time normalized gait cycle.

The group ensemble averaged EMG patterns for all 4 gait speeds are shown in **Fig 3**. As becomes evident from this figure, for most of the 13 muscles the amplitude of activity generally increased with speed.

### Gain functions and gain modules

**Fig 4:**
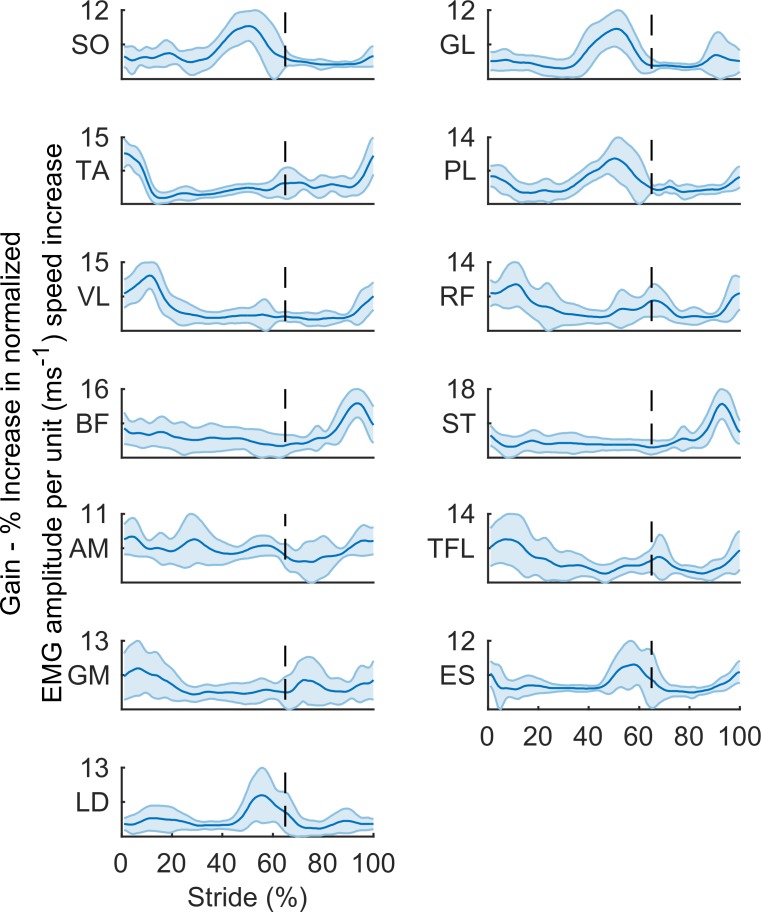
The group averaged (± 1 sd) gain functions that represent the increase in normalized EMG amplitude per unit increase in speed (ms^-1^), for Soleus (SO), Gastrocnemius Lateralis (GL), Tibialis Anterior (TA), Peroneus Longus (PL), Vastus Lateralis (VL), Rectus Femoris (RF), Biceps Femoris (BF), Semitendinosus (ST), Adductor Magnus (AM), Tensor Fascia Latae (TFL), Gluteus Maximus (GM), Erector Spinae (ES), and Latissimus Dorsi (LD). The dotted lines represent swing onset at 65% of the time normalized gait cycle.

Our first aim was to establish if speed related neuromuscular gain is subject to modular control. The group averaged gain functions for each muscle are depicted in **Fig 4**. The average gain function values were positive for all muscles, indicating that increases in gait speed generally resulted in increases in the amplitude of muscle activity, for the entire set of muscles. Visual inspection of **Fig 4** also shows that increases in the amplitude of muscle activity were phase specific, and that the gain functions of particular groups of muscles share similar temporal characteristics.

To test whether the gain functions accurately represented the observed relationship between speed and EMG amplitude, the observed EMG patterns at the 1.00 ms^-1^ and the gain functions for each muscle were combined to reconstruct the group averaged EMG profiles recorded at 0.69, 1.31, and 1.61 ms^-1^. Overall, the quality of the resulting reconstructions was good. Averaged over all muscles, the mean percentage (± sd) of variance in the observed EMG patterns that was accounted for by the reconstruction was 78.9% (± 5.4), 93.3% (± 2.9), and 97.4% (± 1.4), for the EMG profiles recorded at 0.69, 1.31, and 1.61 ms^-1^, respectively. This indicates that the effects of speed on EMG amplitude were reflected accurately in the gain functions.

**Fig 5:**
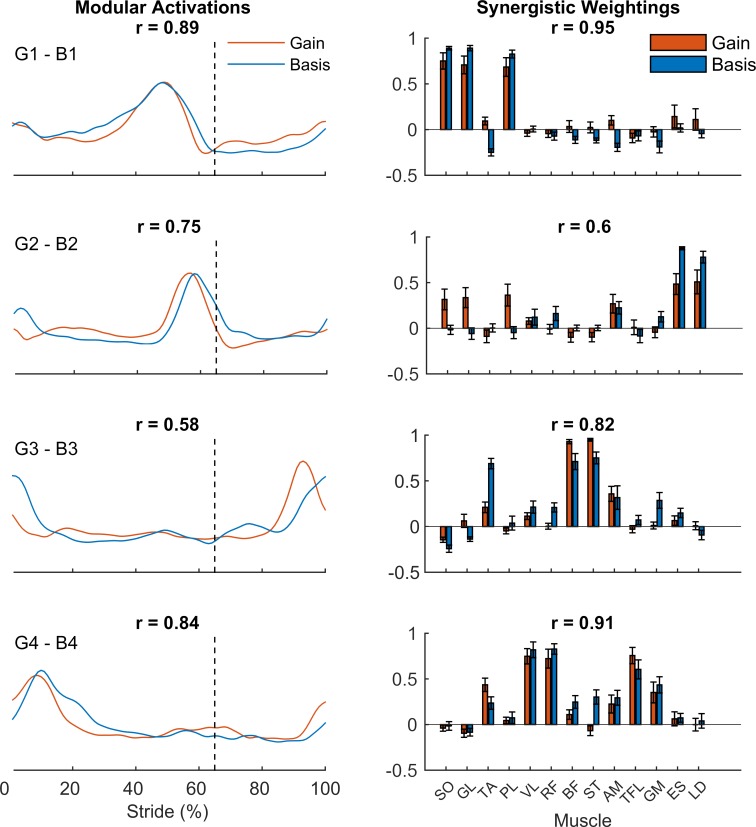
Left column: Modular basis functions (blue, B1-B4) and modular gain functions (orange, G1-G4). In each panel, the correlation R between the corresponding modular basis and gain functions is shown. The dotted lines represent swing onset at 65% of the time normalized gait cycle. Right column: the group averaged synergistic muscle weightings (+1 sd) associated with the modular basis functions (blue, B1-B4) and modular gain functions (orange, G1-G4). In each panel, the correlation between both sets of weightings is indicated as R.

To assess if there is synergistic structure in the neuromuscular regulation of speed, the gain functions were subjected to PCA with varimax rotation to extract 4 modular gain functions and a set of synergistic weightings for each of the functions. The 4 group averaged modular gain functions that were obtained, and their associated synergistic weightings, are presented in the left and right column of **Fig 5**. Averaged over the group, the variance accounted for by the set of modular gain functions was 74.0% (± 1.3%), indicating that there was communality in the gain functions, and that the major portion of variance in the set of 13 gain functions could be accounted for by 4 functions.

### Comparison of modular gain functions and modular basis functions

To determine whether the basis activation of muscles and the speed-related gain are driven by a single synergistic control structure, dimensionality reduction (PCA + varimax rotation) was also performed on the EMG patterns recorded at 1.00 ms^-1^. This resulted in a set of modular basis functions and a set of associated weightings, which are depicted in **Fig 5A** and **Fig 5B** (blue lines), respectively. Averaged over the group, the 4 modular basis functions accounted for 80.1% (± 1.4) of the variance that was apparent in the EMG patterns at 1.00 ms^-1^. This implies that most of the variance in the muscle activity at 1.00ms^-1^ could be accounted for using 4 basis modules.

The number of modular functions that could be grouped using k-means clustering ranged from 11 (component B2) to 13 (component B1, B3 & B4) for the basis modules, and from 9 (component G3) to 13 (component G4) for the gain modules, indicating that the modular basis functions and modular gain functions were representative for the group. Correlations between the matched modular basis functions and modular gain functions were moderate (0.58 and 0.75 for component B3-G3 and B2-G2), and high (0.84 and 0.89 for B4-G4 and B1-G1). This indicates that there were clear similarities in the temporal properties of the basis and gain modular functions.

### Inspection of the synergistic muscle weightings of both types of modular functions

Inspection of **Fig 5** shows that the patterns of synergistic muscle weightings associated with the modular basis and gain functions, generally exhibited similar groupings of muscles. Correlations between the muscle weightings associated with the basis and modular activations were moderate (0.6 for component B2-G2), high (0.82 for B3-G3), to very high (0.95 and 0.91 for B1-G1 and B4-G4), indicating that the matched modular basis and gain functions were driven by very similar synergistic weighting patterns. The muscle weightings associated with B1 and G1 were both dominated by the calf muscles (SO, PL, and GL), whereas weightings on these functions of other muscles were close to zero, resulting in a very high correlation (0.95) between both sets of weightings. The pattern of weightings for modules B2 and G2 showed high weightings for ES and LD on both types of functions, although the calf muscles (SO, PL, and GL) showed clear weightings on the modular gain functions that were not apparent in the weightings on the modular basis functions. Patterns for modules B3 and G3 showed high weightings for the hamstrings (ST and BF), whereas TA weightings were apparent with respect to B3, but not G3. Finally, muscle weighting associated with B4-G4, showed high muscle weightings for the quadriceps group (VF and RF), and to a lesser extend for TFL and GM.

### Reconstruction and prediction of muscle activation patterns

**Fig 6:**
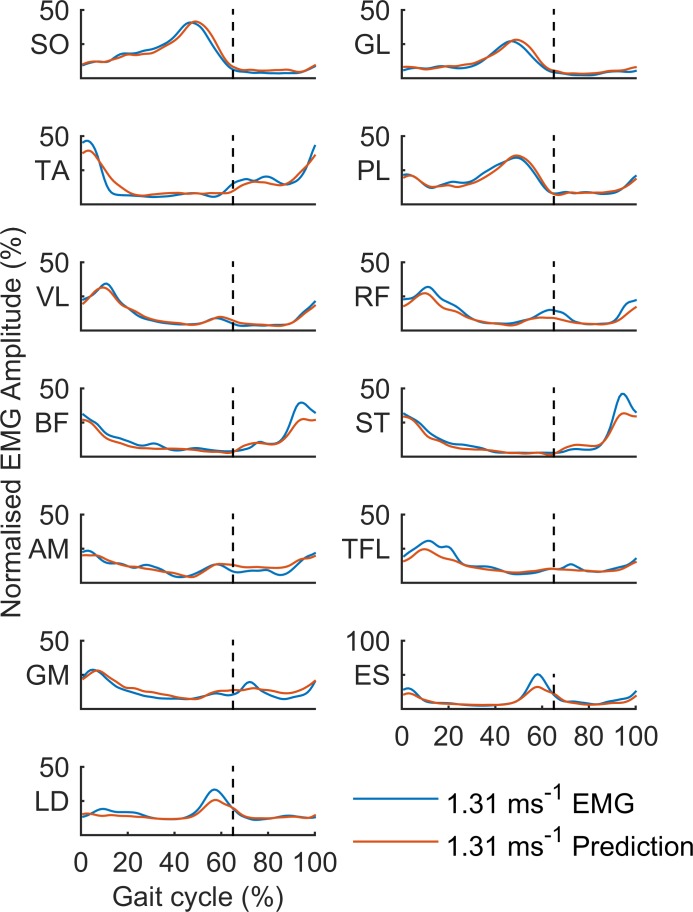
The group averaged EMG patterns at 1.31 ms^-1^ (blue) and the predicted EMG patterns at 1.31 ms^-1^ based on the modular basis functions, the modular gain functions, and their associated muscle weightings. The dotted lines represent swing onset at 65% of the time normalized gait cycle.

To test the robustness of the present approach, we tested (i) the extent to which the averaged EMG profiles of individual participants could be reconstructed using the combined sets of 4 basis and gain modular activations and the associated muscle weightings, and (ii) whether these modular activations and weightings could predict the individual EMG patterns at a speed that was not used in the dimensionality reduction (i.e. at 1.31 ms^-1^). The group average EMG, and predicted EMG per muscle are shown in **Fig 6**. The results confirmed the robustness of the present approach. The percentage variance accounted for by the reconstruction was 93.8% (± 2.1), 92.8% (± 3.1), and 79.7% (± 5.3) for the EMG patterns at 1.61, 1.00, and 0.69ms^-1^, respectively. The predicted EMG patterns at 1.31 ms^-1^ accounted for 92.4% (± 1.5; range between muscles 76.2%–97.9%) of the variance in the recorded patterns at this speed, indicating that the EMG patterns at this speed were predicted quite accurately using the obtained sets of basis and gain modular activations and synergistic weightings.

To determine how well the modular gain functions were capable of predicting novel data of different subjects, we applied a bootstrapping procedure. The average percentage of variance accounted for by combining modular basis functions of n = 6 subjects with the averaged modular gain functions of the n = 7 remaining subject (with 95% confidence interval, CI) was 80.3% (79.6–81.0%), 83.0% (82.5–83.4%), 91.1% (90.6–91.5%), and 67.8% (66.6–69%) for the predictions of EMG profiles at 1.61, 1.31, 1.00, and 0.69 ms-1, respectively.

## Discussion

Previous research has shown that modular activations and (to a lesser extent) their associated synergies show clear similarities over a range of speeds[5,18,20]. However, the established similarities do not necessarily provide evidence that the dynamic regulation of speed itself is under modular control, but instead imply that muscle activations at different speed show a degree of pattern similarity. Here, we elaborated on this work and attempted to separate the ‘pure’ speed effects from the basis activation so as to determine if the phase-specific modulation of muscle output amplitude contains synergistic structure, as was previously implied in the work of Hof et al.[13]. To this end, gain functions were constructed, and their modular properties were assessed and compared to the modular properties of the basis activations of muscles. The results showed that the gain functions displayed a synergistic structure that was associated with clearly distinguishable muscle groups. Furthermore, the modular decompositions were similar for gain functions and the basis activity of muscles, suggesting common modular control for the basic phasing of muscle activity and its modulation by speed. The robustness of the present approach was confirmed, as for the majority of muscles a combined set of modular basis and gain functions could be used to successfully predict muscle activation patterns at a speed (1.31 ms-1) that was not involved in the modular decomposition.

### There is synergistic structure in the speed dependent modulation of muscle activity

The gain functions that were calculated provided a good representation of the phase dependent modulation of muscle output amplitude by speed[11–17]. By modulating the activity at 1.00 ms^-1^ with the constructed gain functions, we were able to reconstruct the EMG patterns at other speeds accurately, explaining 79.7%, 93.8%, and 92.8% of the variance contained in the EMG signals collected at 0.69, 1.61, and 1.31 ms^-1^, respectively. It is worth noting that gain values for the present speed range were mostly positive, and that no paradoxical speed effects (i.e. a decrease in amplitude with increasing speed) were apparent, as was previously reported for speeds slower than 0.28ms^-1^[12].

The ability to reconstruct EMG patterns from the combined set of modular basis and gain functions depended to some extent on gait speed, as the percentage of variance in EMG patterns explained by this set of functions ranged from 79.7% at 0.69ms-1 and 93.8% at 1.61ms-1. This is likely due to the fact that at lower speeds, the signal to noise ratios are typically lower than at higher speeds. More specifically, assuming that the level of noise (or ‘error variance’) apparent in the EMG signal remains approximately constant over speeds, and because the amplitude of the EMG patterns increases with speed[12,13], the relative contribution of ‘systematic variance’ to the observed EMG signals is larger at higher speeds. Because predictions only relate to the systematic part of the EMG pattern variance, prediction quality is likely to better at higher speeds.

Previous studies[18–21] have shown that the activity of large groups of muscles over a range of speeds can be faithfully reproduced through the combined activity of a small number of modular activations. Here, we used a more direct approach to test the synergistic control of speed regulation by applying dimensionality reductions directly to the functions that represent the modulations of muscle activity through speed. The results showed that most of the variance in these functions (74.0%) could be captured using 4 modular gain functions that were associated with clearly distinguishable synergistic muscle weightings. These findings provide direct evidence that the dynamic adjustments made to muscle activity for the regulation of speed, do not depend on individual muscle control, but instead contain synergistic structure.

Inspection of the timing properties of the modular gain functions and their associated synergistic muscle weightings, suggests that the obtained modules reflect specialized biomechanical functions involved in the regulation of gait speed. The activity of the calf muscle group is represented by function G1, with a peak between 40 and 55% of the gait cycle. This muscle group plays a prominent role in propulsion and speed control[17,34,35], as it controls the CoM momentum during single stance, and regulates the temporal (cadence) and/or spatial (length) properties of the step that determine the speed of progression[9,10,36]. Function G2 was associated with activity of ES and LD, and to a lesser extent to the calf muscles, and may serve to accommodate speed-dependent requirements related to swing initiation[17,35,36], and trunk stability during weight shift[37,38]. Function G3 (80–10% of the gait cycle) reflects modulation of activity in the hamstrings (ST and BF) and regulates leg deceleration and a feedforward mechanism to prepare for foot landing during late swing and early stance [16,17,35]. Finally, function G4 (95%-20%, associated with VL and RF, and to a lesser extent TFL and GM), may reflect speed-related control of vertical support and stability during early stance as the vertical excursion of the body’s CoM increases[17,35,39]. Taken together, these findings suggest that the modular gain functions reflect a parsimonious control strategy that translates higher order control signals to specific low level task commands to be implemented for the regulation of gait speed.

### The basic phasing of muscle activity and the regulation of speed are part of a single modular control scheme

One of the questions we wished to address in this study was whether the basic activation of muscles, and the modulation of this basic activity by speed, are controlled by independent networks or if both are controlled by a single modular control structure. The present results clearly favor the latter scenario, as correlations between the matched modular basis functions and modular gain functions were generally high (r = 0.58 to 0.89). Importantly, these functions drive very similar muscle groups as became evident from the high correlations between the associated synergistic weightings of both types of functions (r = 0.6 to 0.95). These combined findings provide direct evidence for the claim made previously that a single set of modular activations controls walking over a range of speeds[18–21]. In this context, it is important to note that basis modules and gain modules appear to represent the same biomechanical subtasks of propulsion (B1-G1), weight shift and swing initiation (B2-G2), leg deceleration (B3-G3), and vertical support and stability (B4-G4). These findings are in line with results from a simulation study by Neptune and co-workers[17], showing that the amplitude of muscle activity changes over a range of speeds, but that the contribution of muscle groups to locomotor subtasks remains invariant over speeds.

The observed similarity between the modular basis and gain functions, and between their respective synergistic weightings, hint at two different scenarios for the modular architecture that is involved in the regulation of gait speed. First, the most parsimonious modular design would involve a single, sparse set of activation patterns, whose modular outputs are scaled approximately linearly to drive a single set of synergistic muscle groups over a broad range of gait speeds[21]. A second scenario is that the modular basis functions and modular gain functions represent higher level and lower level aspects of a hierarchical modular architecture, that together drive an invariant set of task relevant muscle synergies. The lower level modular control may involve the use of task specific afferent information to fine-tune basic modular activations to accommodate phase specific speed requirements. The importance of afferent information for the regulation of specific locomotor subtasks is well established[40–42], and the involvement of afferent mechanisms as part of a less centralized modular architecture has already been hinted at by others[31,43]. This type of hierarchical modular control can be efficient, fast and flexible, and allows higher order modular control to be adapted to a wide range of contexts and task demands. Integration of afferent information in the modular control structure may also be important for adjustments to the relative timing of modular basis and gain activations. Different speeds are associated with different relative durations of stance and swing phases [12,16] and the timing of modular activations needs to be controlled to establish functionally equivalent gait kinematics over a range of speeds[18–20]. In the present study, EMG data were time normalized separately for stance and swing phases to make possible a meaningful analysis in terms of gain functions, and as a consequence all information about possible phase shifts in the modular activations was neutralized. Nevertheless, it is well possible that the timing of the here presented modular (basis and gain) activations is subordinate to a temporal controller that is informed by afferent information on gait phase transitions[16,44].

With regard to the modular control that may govern adaptive walking, it is important to acknowledge that flexible recruitment of a single set of modular activations and synergistic weightings to accommodate speed requirements, needs to involve additional layers of control e.g. to induce phase shifts[19,20], a speed-dependent scaling of synergistic weights[18], or the construction of an additional set of functions and weights as in the present study. An important advantage of approaches like that of Gonzalez-Vargas et al.[18] and the approach described in the present paper, is that it is made explicit which elements of the modular control structure are sensitive to changes in speed. It can be argued that the here proposed mediation through an extra control layer, involving modular gain functions and their associated synergistic weights, implies an increase in the complexity of modular control. However, the observed similarities between modular gain and basis functions and their associated sets of weights, as well as the functional relatedness that is implied in this, suggests that the combined sets of functions and their synergistic weights are reflective of a parsimonious form of adaptive control.

### Prediction of muscle activation patterns for a given speed

Since the modular gain functions represent the linear change in activity as a function of speed, speed can be used as an input to these functions to not only reconstruct, but also predict muscle activations at any given speed. We exploited this property to test the robustness of the presently used approach and predict muscle activation patterns for a speed (1.31 ms^-1^) that was not involved in the construction of the (modular) gain functions. Overall, the combined set of modular basis and gain functions provided accurate predictions of the activations of individual muscles, as on average 92.4% of the variance in the recorded EMG patterns at 1.31ms^-1^ was accounted for by the predictions.

The good predictive properties of the combined set of modular functions and associated synergies imply that the here used modular decomposition provide an accurate representation of the basic phasing of muscle activity, and the phase-specific modulation of this basis activity by gait speed. Although recent research has shown that similarly good predictions can be produced through speed-dependent modifications in the synergistic weights that drive a single set of modular basis functions[18], the present simulations elaborate on these findings and show that a modular decomposition that is aimed directly at the speed-dependent modulation of muscle-output amplitude (i.e. the modular gain functions), provides additional evidence that for the claim made previously [18–21] that a single set of modular activations controls walking over a range of speeds.

The finding that muscle activations can be predicted for a given speed using a set of (modular) gain functions, may have interesting applications for research on neuromuscular control in special populations, e.g. in patients or frail elderly. As it is often difficult to exert experimental control over the potentially confounding effects of gait speed, the approach used here may be used to compare groups at a single, simulated speed, so as to detect differences in the patterning of muscle activity that that are not related to speed[23]. Overall, the present results also suggest that a representation of the effects of naturally varying parameters (e.g. cadence, loading) as time varying functions, and the assessment of their modular properties, may provide interesting opportunities for studying the flexibility in the modular control of gait. These findings may further inspire work on flexibility in the modular control of gait in populations with impaired walking ability[21,45].

## Supporting Information

S1 Data The worksheets ‘EMG 1.61 ms-1’, ‘EMG 1.31 ms-1’, ‘EMG 1.00 ms-1’, ‘EMG 0.69 ms-1’ show group-averaged time-normalized EMG data at 1.61 ms^-1^, 1.31 ms^-1^, 1.00 ms^-1^, 0.69 ms^-1^ gait speed for the following 13 muscles: Soleus (SO), Gastrocnemius Lateralis (GL), Tibialis Anterior (TA), Peroneus Longus (PL), Vastus Lateralis (VL), Rectus Femoris (RF), Biceps Femoris (BF), Semitendinosus (ST), Adductor Magnus (AM), Tensor Fascia Latae (TFL), Gluteus Maximus (GM), Erector Spinae (ES), and Latissimus Dorsi (LD). The worksheet ‘Gain functions’ shows group-averaged gain functions for all 13 muscles. The worksheets ‘Gain modules’ and ‘Basis modules’ show the four group-averaged gain modules and the four group-averaged basis modules that resulted from the clustering protocol. The worksheets ‘Gain Synergies’ and ‘Basis Synergies’ show the four group-averaged gain synergies and the four group-averaged basis synergies for all 13 muscles that resulted from the clustering protocol. The worksheets ‘Reconstruction 1.61 ms-1’, ‘Reconstruction 1.00 ms-1’, ‘Reconstruction 0.69 ms-1’ show the group-averaged reconstructed profiles at 1.61 ms^-1^_,_ 1.00 ms^-1^, and 0.69 ms^-1^ for all 13 muscles. The worksheet ‘Prediction 1.31 ms-1’ shows the group-averaged predicted profiles at 1.31 ms^-1^ for all 13 muscles.
(XLS)
