# Correction: Synergistic Structure in the Speed Dependent Modulation of Muscle Activity in Human Walking

**DOI:** 10.1371/journal.pone.0154910

**Published:** 2016-04-28

**Authors:** The PLOS ONE Staff

The second author’s name is spelled incorrectly. The correct name is: Claudine J. C. Lamoth. The publisher apologizes for the error.
